# Optimization of the mechanical properties of polyester/coconut shell ash (CSA) composite for light-weight engineering applications

**DOI:** 10.1038/s41598-022-26632-x

**Published:** 2023-01-19

**Authors:** O. O. Daramola, A. A. Akinwande, A. A. Adediran, O. A. Balogun, J. L. Olajide, K. J. Adedoyin, B. O. Adewuyi, T. C. Jen

**Affiliations:** 1 Department of Metallurgical and Materials Engineering, Federal University of Technology, Akure, 340001 Nigeria; 2 Department of Mechanical Engineering, Landmark University, PMB, Omu-Aran, 1001 Kwara State Nigeria; 3 Department of Mechanical and Automation Engineering, Tshwane University of Technology, Pretoria, South Africa; 4 Department of Nuclear Safety, Physical Security and Safeguards, Nigerian Nuclear Regulatory Authority, Abuja, Nigeria; 5 Department of Mechanical Engineering Science, University of Johannesburg, Johannesburg, South Africa

**Keywords:** Engineering, Materials science

## Abstract

The mechanical properties of coconut shell ash (CSA) reinforced polyester composite have been optimized. Various test specimens were developed by dispersing 10, 20, 30 and 40 wt.%, of CSA in unsaturated polyester resin in decreasing particle sizes of 40, 30, and 20 µm in an open mould using hand lay-up technique. Tensile, flexural, and impact strengths, as well as tensile and flexural moduli and Shore D hardness of all test samples were determined. The results showed that 10–20 wt.% CSA increased tensile, flexural, impact strengths and flexural modulus for all particle sizes, but 30–40 wt. % CSA engendered depreciation in corresponding performance. For all particle sizes, 10–40 wt. percent CSA resulted in an increase in tensile strength, whereas 10–40 wt. percent resulted into a linear increase in Shore D hardness. Further observation portrayed that in each case, the finest CSA (20 µm) have the optimum result. Statistical analysis carried out on experimental outcomes confirmed the experimental variables (particle proportion and sizes) to be significant. From the surface plot, the strength responses revealed more dependence on the individual variables than their interactions. Regression models developed for individual responses are termed statistically fit in representing the experimental data.

## Introduction

On account of their light weight, corrosion resistance, and affordability, polymers and their composites have developed a reputation as engineering materials. They have gained application as vehicle and aerospace component parts, as well as in biomedical applications. Polycarbonates (PC), polyamides (PA), polyurethane (PU), which are thermoset plastics, and acrylonitrile butadiene, an amorphous thermoplastic polymer, are the most commonly utilized polymeric materials in automobiles^1^. The aforementioned thermoset materials are more expensive, comparing their cost with that of polyester resin, though they possess superior properties. Polyester is regarded as a worthy material for automotive design due to its inexpensive cost^2,3^.

In recent studies, additives are sourced and incorporated into polymer matrices using various processes to improve their properties^4–7^. Ceramic or synthetic fibers or particles are often being utilized as the additives with proven results^7–10^. Natural-derived ones, on the other hand, are more cost-effective. Several studies have been conducted investigating the use of powders and ashes obtained from agricultural waste and by-products as matrix fillers^11,12^. Their integration in polymer was acclaimed as an effective way to recycle while also generating environmentally friendly materials. This reduces the environmental impact of the original wastes while also promoting biodegradable and environmentally friendly composites^13,14^.

Among the studies on the usage of particles in polymer reinforcement is that of Daramola and Akintayo^15^. Green silica generated from rice husk ash was injected into epoxy matrix at 0.5, 1, 2, 3, 4, 5, and 6 wt.% in their investigation. The particles were found to improve the composite's modulus of elasticity, with an optimum value at 2 wt.%. At 1 wt.% addition, peak flexural strength was achieved. The data also demonstrated a gradual increase in hardness and flexural modulus between 0.5 and 3 wt.%, with a deterioration in property values at 4–6 wt.%. Similarly, Oladele et al.^16^ accessed the effects of palm kernel shell ash on the characteristics of epoxy as a material for vehicle bumpers. The ash particles were introduced in doses of 2, 4, 6, 8, 10, 15, and 20 wt.%. At 2–8 wt.% inclusion of the particles, there was an improvement in tensile and flexural strengths. Water absorption rose as filler dosage increased, while wear resistance increased linearly from 2 to 20%. The end result revealed that better epoxy composites could be made by employing agro-based filler. Achukwu et al.^17^ found that palm kernel shell powder at a concentration of 5% increased the tensile strength of epoxy while also increasing the tensile modulus up to 40 wt.%. 10% egg shell powder loading was demonstrated to give peak impact strength and hardness in the polypropylene matrix produced by Mchuaran et al.^18^. Tensile and flexural moduli of polypropylene reinforced with the filler increased from 5 to 55 wt.%. While tensile strength decreased with increment in filler doses from 5 to 55 wt.% filler, flexural and impact strength appreciated between 5 and 40 wt.%.

Groundnut shell powder is another type of filler reinforcement. Groundnut shell powder was blended at 5 to 25 wt.% (in 5% increments) in high-density polyethylene by Jacob et al.^19^. At particle loadings of 20, 25, 20, and 20%, peak tensile strength, hardness, elastic modulus, and modulus of rupture were achieved, correspondingly. Usman et al.^20^ studied the influence of pulverized shell powder on the mechanical properties of polyethylene matrix at particle loadings ranging from 5 wt.% to 25 wt.% (in 5 wt.% increments). While tensile strength was observed to improve linearly between 5 and 25 wt.% of the filler, maximum elastic modulus was reported to be generated by 20 wt.% of the ash. Other studies in this area can be found in the literatures^21–23^.

The results of research on polyester composites have been presented. Imoisili et al.^24^ investigated the use of cocoa pod ash as filler in polyester at concentrations of 1, 2.5, 5, 7.5, and 10%. Tensile and specific moduli were revealed to rise between 1 and 2.5 wt.%, but elongation at break was noted to decrease as the ash dosage was increased. Imoisili et al.^25^ studied the role of NaOH and silane treated rice husk flour (RHF) in polyester composites using rice husk flour. The filler dosages were 5, 10, 20, 30, and 40% by weight. Tensile strength rose between 5 and 10 wt.% RHF for NaOH treated composite and 5–20 wt.% for silane treated. Furthermore, hardness was boosted between 5 to 10 wt.% of the filler for both types of treatment. According to the research, treated rice husk flour has the ability to improve the characteristics of polyester by 10% to 20%. Similarly, Achukwu et al.^26^ had achieved the improvement of elastic stiffness and flexural rigidity of polyester with powder derived from flamboyant (*Delonix regia*) pod.

Hassan et al.^27^ investigated the effect of egg shell particles (both uncarbonized and carbonized) on some mechanical properties of polyester matrix. As reported, hardness and compressive strength of the polyester was improved upon between 10 and 50 wt.% of the fillers with carbonized performing better. 20 wt.% of the filler achieved peak flexural strength with the carbonized yielding better result. Other study involved in particulate filling of polyester are showcased by Ifeyinwa et al.^28^; Bolasodun et al.^29^.

Majority of studies on particulate-reinforced polymers accessed the effect of the filler proportion on the properties of the polymer material, while some examined the effect of the proportion and particle sizes. However, there are fewer studies which modeled the properties of polymer composites as function of particle proportion and particle sizes. Similar optimization and simulation study can be found in literatures^30,31^ in which the structural responses in terms of frequency and deflection of luffa cylindrical fiber-reinforced epoxy composite was computed and simulated by finite element modeling. Also, the acoustic responses of natural fiber reinforced polymer nanocomposite was simulated by ref^32^. Other related works involving simulation, modeling and optimization can be found in literatures^33–37^. Therefore, the effects of varied proportions and particle sizes of coconut shell ash on polyester/coconut shell ash composites were investigated in this study with the goal of modeling the experimental results using a response surface technique. The choice of coconut shell is on the premises of its availability and abundance in the area where the research was carried out.

## Materials and methods

**Figure 1:**
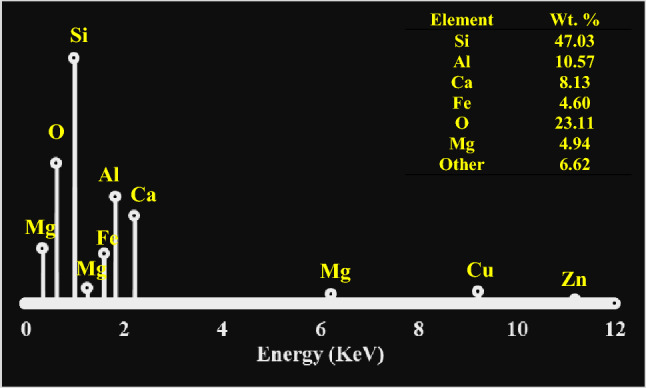
Elemental composition of the coconut shell ash as obtained by energy dispersive spectrometer (EDS)

The elemental composition of CSA is presented in Fig. 1 while Table 1 revealed its chemical composition.

**Table 1:** Chemical composition of coconut shell ash (CSA).

Chemical	Al_2_O_3_	CaO	SiO_2_	K_2_O	Na_2_O	MgO	Fe_2_O_3_	LOI	Other
Content	19.50	8.90	44.7	0.34	2.40	5.10	7.50	6.10	5.46

Coconut shells sourced locally were washed, dried and broken into smaller pieces. The products obtained were pulverized to increase the surface area and placed in a muffled furnace. The furnace was operated to 700 °C and maintained for 5 h before allowing the ashes to cool to room temperature. The ashes were then sieved to 40, 30 and 20 µm passes and collected for the experimental process. Figure 1 reveals the elemental composition of the depicting major elements to be Si, Al, Ca, Fe, similar to the elemental composition of CSA showcased in the previous studies^38,39^. Table 1 presents the chemical distribution within the ash. Its major constituents are silica and alumina which has been confirmed as major reinforcing constituents in ashes derived from agro-by-products^40^. Hand-lay-up process was engaged in the development of the composite samples. CSA/polyester composites were produced by mixing 10, 20, 30, and 40 wt.% of CSA particulate in particles sizes of 40, 30 and 20 µm with polyester matrix in various test moulds. After homogeneous mixing, the polymerization process was initiated with addition of catalyst and accelerator. The different compositions of the polyester composites were cured at room temperature conditions for one hour, and carefully de-moulded. The produced composites were further allowed to cool in air (room temperature) for 48 h.

## Property evaluation

The properties of the CSA/polyester composites were assessed for the purpose of observing the influence of the CSA addition on the polyester matrix. The procedures taken in accessing each property response are indicated in this section.

### Tensile properties of CSA/polyester composite

Composite samples (50 × 5 × 10 mm) were prepared and subjected to tensile test employing Universal testing machine (Instron 3369-E). Load of 100 N was applied at strain rate of 3 mm/min. The samples were carefully placed between the teeth (or grip) of the machine, to be automatically pulled till failure. The process was repeated for all samples (including control) at different particle doses and sizes. Suitable software incorporated with the machine read the tensile strength, and tensile modulus values of the samples. The test was carried out in triplicates for each mix proportion and average values were taken as the absolute values. Recordings were made appropriately^42^.

### Flexural properties of CSA/polyester composite

In line with ASTM D790^41^, bending test was effected on the developed composite specimen of dimension 150 x 50 x 3 (mm^3 ^) at cross speed of 0.3 mm/min and   strain rate of 10^-3^/s. The test was carried out in triplicates for each mix proportion and average values were taken as absolute values.

### Hardness of CSA/polyester composite

The hardness tests were carried out on the composite samples with the aid of Shore D hardness tester, using a load of 100 g, at a holding time of 15 s. Four different indentations were made on each sample, and the average of the value was recorded^43^.

### Impact strength of CSA/polyester composite

The notched Izod impact test was conducted in accordance with ASTM D256–10 (2018)^44^. The test was carried out using a Hounsfield balanced impact testing machine, (3915-H10-3). Impact test samples (dimension of 64 × 11 × 3 mm) were notched at the center. Samples were placed horizontally on the machine, maintaining a distance of 60 mm between lines of supports. The test samples were placed in a cantilever position, clamped upright with a V-notch at the level of the top of the clamp. The machine pendulum hit the test piece and was allowed to fall freely to a fixed height.

## Results and discussion

Results obtained at each dosage addition was compared with the result of the pure polyester matrix to assess the percentage contribution realized at each dosage addition of CSA addition.

### Tensile strength and tensile modulus

**Figure 2:**
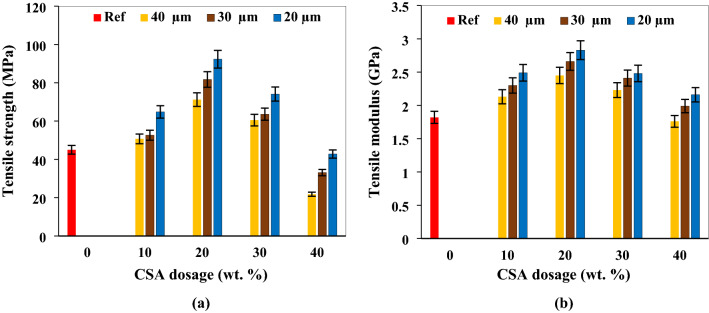
Influence of CSA proportion and size on (**a**) tensile strength (**b**) tensile modulus.

Figure 2A featured the tensile strength of the CSA/polyester composite at varying proportion and particle sizes of filler. In this study, an uptrend in tensile strength was observed between 10 and 20 wt.% CSA for all particle sizes. Appreciation in strength based on increasing volume of particulates is associated with adequate dispersion of the particles within the matrix and strong particle–matrix bond. Alias et al.^45^; and Achukwu et al.^46^ linked the feat to good filler-filler interaction. Hassan et al.^27^ infused maize stalk ash as reinforcement in polyester matrix. 20 wt.% maize stalk ash yielded optimum strength which was ~ 57.1% improvement in strength. Compared with this study, 20 wt.% (particle size 20 µm) spawned 90.3% accretion in strength depicting CSA to be a good reinforcement, hence, performing better than maize stalk ash. Similar result was reported by Atuanya et al.^47^ in which bean pod ash was infused at 5 to 30 wt.% in low density polyethylene matrix. Peak tensile strength was realized at 20 wt.% of the particulate. Investigations carried out by Ojha et al.^48^ revealed infusion of carbonized coconut shell particles in epoxy at 5, 10, 15 and 20 wt.% result of which pinpointed that peak strength was attainable at 15 wt.% of the particulate. However, the outcome presented in present investigation stands contrary to the findings of Agunsoye et al.^49^ as 5, 10, 15, 20 and 25 wt.% coconut shell particles led to progressive reduction in strength as observed in their report.

Disparity in the particle sizes was revealed to influence the tensile properties of the composite (Fig. 2). For particle size of 40 µm, 10 and 20 wt.% CSA ensued 4.5 and 46.8% enhancement relative to the reference mix containing 0 wt.% CSA. Corresponding dosage for particle size of 30 µm resulted in 8.5 and 68.5% improvement while particle size of 20 µm showed 33.6 and 90.3% increment in strength in comparison with the reference mix. From this result, reduction in particle sizes amounted to higher strength. The feat is on account of larger surface area possessed by the smaller particles, hence there is increased area of stress distribution within the matrix, thereby enhancing the strength. Atuanya et al.^50^ introduced snail shell particles in low density polyethylene at particle sizes of 500, 250, 125 and 75 µm in the proportion of 5, 10 and 15 wt.%. Response of the tensile strength to the variables showed appreciation in strength as proportion increased while reducing size resulted in better strength performance. Highest strength was recorded when 15 wt.% of the particulate of the smallest particle size was infused. This observation agrees with the turn of events in this study as peak tensile strength was attained when the smallest particle size was infused, though, at 10 wt.%. 10 wt.% assimilation of rice husk ash in polyethylene matrix gave maximum tensile strength in the work of Atuanya et al.^51^ which is in tandem with the optimum result showcased in this study.

In present study, 30 and 40 wt.% CSA led to sequential decrease in the response imputable to weaker interfacial bonding and particle coagulation. Comparable findings were reported by Zhang et al.^52^ in which the blend of rice husk biochar led to consecutive enhancement of tensile strength of high-density polyethylene matrix at 10, 20, 30 and 40 wt.%. Zhang et al.^52^ further reported that the inclusion of 50, 60 and 70 wt.% engendered continual decrease in strength. It is observed that increasing dosage of the particulate between 10 and 30% promoted progressive enhancement of the tensile modulus (Fig. 2b). This is due to good interfacial bonding between the particulate and the polymer matrix. Dispersion of the particulate within the polymer matrix stood as obstacle to chain slide during deformation, eventually increasing stiffness. The result showcased in present study is consistent with the result depicted by Atuanya et al.^47^ as increasing proportion of CSA from 5 to 30% enhanced the tensile modulus. In the same way, mahogany filler added to polyethylene in the dosage of 5 to 35 wt.% (at 5% interval) resulted in progressive increase in tensile modulus^53^. The research carried out by Atuanya et al.^50^ where snail shell particles were blended with low density polyethylene engender enhancement of the modulus which conforms with the modulus pattern in the present study. Furthermore, the snail shell particles were introduced at varying sizes and it was revealed that particles with the lowest size yielded best performance further corroborating our investigation. The findings of Kamalbabu and Kumar^54^ also confirmed the fact that lower particle sizes outperformed larger particle sizes yielding better performance. Due to the small sizes, they are able to effectively fill pores within the matrix, leading to lower spacing, hence improvement of stiffness.

### Flexural strength and flexural modulus

**Figure 3:**
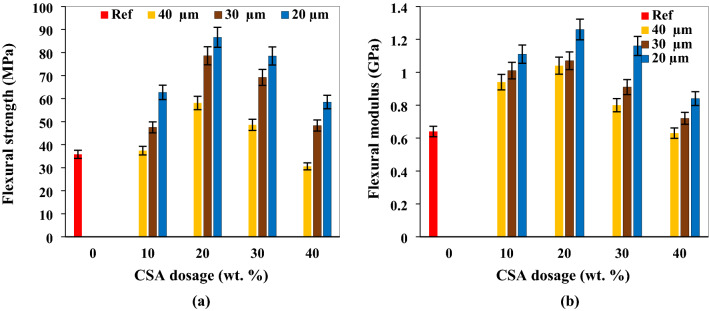
Influence of CSA proportion and size on (**a**) flexural strength (**b**) flexural modulus.

The flexural strength and flexural modulus of prepared composite are presented in Fig. 3a and Fig. 3b respectively. Increment in CSA dosage between 10 and 20 wt.% elicited persistent improvement in the flexural/bending strength and flexural modulus for all particle sizes. The appreciation is linked to lower void content and good stress transfer between matrix and fillers. Further observation reveals smaller particle sizes gave better strength and modulus enhancement owing to interfacial bonding between lesser particles and the polyester matrix. Additionally, the boost in strength and modulus is triggered by particulate dispersion within the matrix and enhanced particle–matrix interaction which enabled even stress distribution.

The showcased outcome, as presented in Fig. 3(a,b), is epitomized in Ojha et al.^48^; an investigation which studied effect of carbonized coconut shell filler on mechanical properties of epoxy resin. Flexural strength and flexural modulus was enhanced progressively up to 20 wt.% of the filler. The result demonstrated 74.4% enhancement at 20 wt.% when compared with the pure epoxy. This equally agrees with Sindhu and Chouhan^55^; Sahai and Mahanwar^56^) as optimal enhancement was achieved on injection of 20 wt.% coconut shell charcoal and 20 wt.% fly ash respectively. Atuanya et al.^47^ introduced bean pod ash filler in low density polyethylene at 5, 10, 15, 20, 25 and 30 wt.%; meanwhile, 20 wt.% of the filler dosage yielded optimum enhancement in flexural strength. The authors attributed the increment in strength between 0 and 20 wt.% to the particles interfering in the mobility and deformability of the matrix.

In the present investigation, 10, 20, and 30 wt.% CSA dosage for particle size 40 µm resulted into 4.4, 62.2, and 35.8 enhancement respectively with respect to reference mix, though 40 wt.% led to a decrease of 14.5%. For particle size 30 µm, 10, 20, 30 and 40 wt.% yielded 32.7, 120.0, 93.3 and 34.9% improvement respectively. Also, 20 µm particle size of CSA at 10, 20, 30 and 40 wt.% led to 75.1, 141.1, 119.3 and 63.4% increment in flexural strength compared with the reference mix. This therefore inferred that as particle size reduces, there is increment in strength. Proportions of 30 and 40 wt.% in the polyester matrix kindled sequential decrease in flexural strength and flexural modulus for all particle sizes. The decrease is ascribed to clustering of particles at higher proportion. The clusters serve as point of stress concentration during loading; effect of which kindled strength and modulus decrease. At higher dosage, there is higher ratio of filler to porosity and as such clustering of particles ensued at varying degrees. Consequent upon that, there is stress concentration at particulate/matrix periphery lessening the potency of the interaction between particles and matrix. The implication of this reflects in the debonding of the particulate/matrix region leading to lowering of strength and modulus. A similar observation is reported by Zhang et al.^52^ in which rice husk biochar introduced in high density polyethylene provoked strength enhancement from 10 to 50 wt. fraction beyond which there was a reduction at 60 and 70% wt. fraction. Additional scrutiny revealed higher strength as particle size reduced for all proportions. Conversely, the result presented in this study is contrary to the outcomes expounded in Doan et al.^57^ and Kolawole et al.^58^. In the work of Doan et al.^57^; 30, 40, 50 and 60 wt. fraction of rice husk filler of sizes 500–850, 350–500 and 180–350 µm resulted in linear decrease in strength at increasing filler loading. Lesser particle sizes which ensued good interfacial bonding between filler and matrix could be the reason for the discrepancy between present study where particles sizes of 40, 30 and 20 µm was adopted compared with the investigations carried out by Doan et al.^57^. The same reason goes with Kolawole et al.^58^ who utilized dates pal pits particulate of sizes 300 and 150 µm between 10 and 50 wt. fractions.

The finding presented in this study as regards effect of particle size of the composite is exemplified in Nwanonenyi et al.^59^. In their study, periwinkle shell powder in mixed with low density polyethylene at filler loading of 0 to 30 wt.% for particle sizes 150, 125 and 75 µ enkindled higher strength as particle sizes reduced for each corresponding proportion of the filler, even as corroborated by Omah et al.^60^.

### Impact strength and hardness

**Figure 4:**
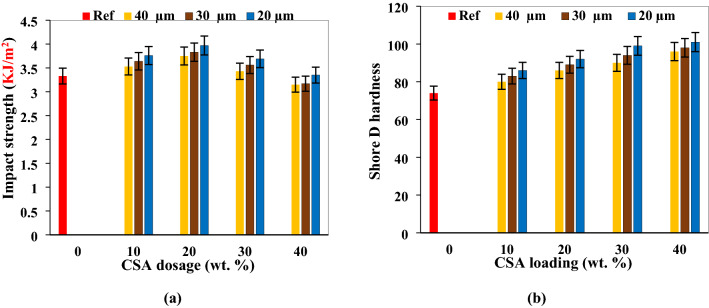
Influence of CSA proportion and size on (**a**) impact strength (**b**) shore D hardness.

Impact strength is influenced by inherent toughness attribute of the reinforcing filler, quality of particulate/matrix interface region and frictional work required in the debonding of the particles from the matrix^47^. The impact strength of the developed composite is dependent on proportion of the CSA and the particle sizes (Fig. 4a). Incorporation of 10 and 20 wt.% CSA ensued strength enhancement for all particle sizes, associated with the ability of the particulate filling voids inherent in the matrix. This finding is similar to the findings of Qian^61^ who evaluated the impact strength of polylactic acid (PLA) strengthened by bamboo biochar particles. In his study, maximum strength was attained when 5 wt.% of the biochar was added. The feat was linked to the filling of the gaps within the PLA matrix enhancing resistance to crack propagation. Ipilakyaa et al.^62^ associated the increase in impact strength to increased elasticity of the matrix enhancing resistance to deformation.

As observed in Fig. 4a, addition of CSA beyond 20 wt.% ensued steady decrease in strength down to 40 wt.%. This experience is epitomized in literatures refs 46, 58, 63. The decrease in strength is associated with weak interaction between the particulate filler and the matrix during loading. Olaitan et al.^63^ linked the depreciation to reduction in the mobility and freeness of the polymer chains evoking decrease in high strain absorption capacity of the composite. Atuanya et al.^51^ reported decrease in the resistance on infusion of rice husk filler in polyethylene matrix between 10 and 35 wt.% associated with poor wetting between the filler and the matrix. Clustering and particle agglomeration are another point contributing to lowering of strength. Sites of agglomeration are sites of stress concentration during deformation which eventually weakens the matrix resistance to high strain deformation.

It is further observed that finer particle sizes of the  CSA outperformed the larger sized particles. For instance, 10 and 20 wt.% CCA yielded 6.0 and 12.6% increase in impact strength for particle size 40 µm CSA when compared with the reference mix. 30 µm CSA led to 9.3 and 15.0% enhancement in strength for the same proportion relative to the reference mix. Similarly, 20 µm CCA exhibited 12.9 and 19.2% improvement for the same proportion. Smaller size particulate fillers manifest better interfacial adhesion with the matrix enabling even or uniform stress distribution. This finding is in tandem with the reports of Atuanya et al.^50^; Kolawole et al.^58^ and Genesca et al.^64^ as smaller particle sized fillers outperformed the larger sized fillers. It is therefore affirmed that CSA not more than 20 wt.% results in enhancement of impact strength of  CSA/polyester composite. Particle size of 20 µm outperformed 30 and 40 µm CSA between 10 and 40 wt.%.

From Fig. 4b, it is depicted that increasing proportion of CSA led to increase in hardness for all particle sizes. The result of Hassan et al.^65^ corroborates the outcome of this study as increasing egg shell particulate loading provoked progressive rise in hardness. Equally, Atuanya et al.^47^ reported that rising bean pod ash particulate engendered progressive enhancement in hardness value. Atuanya et al.^51^ added rice husk filler to polyethylene matrix and observed progressive increase in hardness with increment in filler proportion from 5 to 35% (at 5% interval). Also, groundnut husk particulate infusion in polyethylene by Azeez et al.^66^ revealed consecutive improvement in hardness as filler proportion increased from 5 to 35% (at 5% interval). Same feat was observed in the findings of Hassan et al.^65^; Ahmed et al.^67^; Balogun et al.^68^. The achievement is associated with the hardness of particles owing to the silica and alumina content (Table 1).

Effect of the particle sizes on hardness show that lower particle size performed better in that particle size of 20 µm outperformed 40 and 50 µm for same dosage of CSA filler. The observation is also in tandem with highlighted results presented by Kolawole et al.^58^; Nwanonenyi et al.^67^ and Omah et al.^60^.

### Statistical analysis

A, B, TS, TM, FS, FM, IS, HD stands for particulate proportion (%), particulate size (µm), tensile strength (MPa), tensile modulus (GPa), flexural strength (MPa), flexural modulus (GPa), impact strength (KJ/m^2^), shore D hardness respectively.

Experimental outcomes for each of the response under evaluation were subjected to statistical analysis involving analysis of variance (ANOVA) and response surface analysis. The analysis was undertaken using design expert 13 software. ANOVA was achieved at 95% confidence level and 5% significance. Via this process, the significance of the independent variable: particulate proportion (A) and particulate size (B) were examined for the purpose of accessing their contribution to the magnitude of the responses. The responses for the experimental outcomes against the combination of the parameters are presented in Table 2.

**Table 2:** Experimental outcomes involved in the statistical analysis.

Runs	Variables		Responses					
	A (%)	B (C)	TS	TM	FS	FM	IS	HD
1	10	40	50.7	2.13	37.4	0.94	3.53	80
2	20	40	71.2	2.45	58.1	1.04	3.75	86
3	30	40	60.5	2.23	48.6	0.8	3.43	90
4	40	40	21.8	1.76	30.6	0.63	3.15	96
5	10	30	52.6	2.3	47.5	1.01	3.64	83
6	20	30	81.7	2.66	78.6	1.07	3.83	89
7	30	30	63.6	2.41	69.2	0.91	3.56	94
8	40	30	33.1	1.99	48.3	0.72	3.17	98
9	10	20	64.8	2.49	62.7	1.11	3.76	86
10	20	20	92.3	2.83	86.6	1.26	3.97	92
11	30	20	74.1	2.48	78.5	1.16	3.69	99
12	40	20	42.8	2.16	58.5	0.84	3.35	101

#### Analysis of variance

The ANOVA result on tensile strength and modulus is showcased in Tables 3 and 4. The model presented in Eq. 1 and 2 for the tensile strength and modulus are termed significant since p value is < 0.05. The experimental inputs of particle dosage (A) and particle size (B) are also termed significant since *p* < 0.05 in the two cases. Cross interaction between the variables (AB) is insignificant; same with square interaction *B*^2^. Meanwhile square interaction *A*^2^ has significant contribution on the both responses. Coefficient of determination (*R*^2^) for the regression models developed for tensile strength and tensile modulus are 0.9730 and 0.9544, hence, the model (Eq. 1, 2) are statistically fit to represent the fitted data. Contribution of inputs A and B are 24.77 and 13.58% for tensile strength, 26.07 and 25.48 for tensile modulus. This implies that particle dosage/proportion had higher contribution on tensile strength and modulus as compared with particle sizes. Square interaction *A*^2^ shared highest contribution in each case with values of 58.61 and 43.73% respectively.

1 $$\begin{aligned} {\text{TS}} = & {44}.{7167} + {6}.{7373} {\text{A}}{-} {1}.{9225} {\text{B}} - 0.{148}0 {\text{A}}*{\text{A}} + 0.0{2}0{3} {\text{B}}*{\text{B}} - 0.00{66} {\text{A}}*{\text{B}}, \\ {\text{R}}^{{2}} = & \, 0.{973}0 \\ \end{aligned}$$

2 $$\begin{aligned} {\text{TS}} = & {2}.0{429} + 0.0{799} {\text{A}} - 0.00{33} {\text{B}} - 0.00{19} {\text{A}}*{\text{A}}{-} \, 0.000{2} {\text{B}}*{\text{B}} + 0.0000 {\text{A}}*{\text{B}}, \\ {\text{R}}^{{2}} = & \, 0.{9544} \\ \end{aligned}$$

**Table 3:** ANOVA result on tensile strength (TS).

Source	SS	df	MS	*F* value	*p* value	% Contribution
Model	4363.90	5	872.78	43.28	0.0001	97.30
*A*-particle dosage	1111.12	1	1111.12	55.10	0.0003	24.77
*B*-particle size	609.00	1	609.00	30.20	0.0015	13.58
AB	4.36	1	4.36	0.2160	0.6585	0.10
*A*^2^	2628.48	1	2628.48	130.33	< 0.0001	58.61
*B*^2^	10.94	1	10.94	0.5422	0.4893	0.24
Residual	121.00	6	20.17			
Cor total	4484.90	11				

*SS* sum of squares, *MS* mean square.

**Table 4:** ANOVA result on tensile modulus (TM).

Source	SS	df	MS	*F* value	*p* value	% Contribution
Model	0.9045	5	0.1809	25.11	0.0006	95.44
*A*-particle dosage	0.2470	1	0.2470	34.29	0.0011	26.07
*B*-particle size	0.2415	1	0.2415	33.53	0.0012	25.48
AB	2.500E-06	1	2.500E-06	0.0003	0.9857	0.00
*A*^2^	0.4144	1	0.4144	57.53	0.0003	43.73
*B*^2^	0.0015	1	0.0015	0.2088	0.6638	0.16
Residual	0.0432	6	0.0072			

*SS* sum of squares, *MS* mean square.

Analysis of variance on flexural strength and modulus of the composite are highlighted in Tables 5 and 6. The model terms, for flexural strength and modulus are termed significant (*p* < 0.05). Particle dosage (A) showed no appreciable influence on flexural strength, meanwhile particle size showed appreciable contribution. Also, the two input parameters contributed significantly on the flexural modulus. Cross interaction between the two inputs and interaction *B*^2^ are observed to be inconsequential in the case of the two responses while the square interaction *A*^2^ showed appreciable influence on the responses. The coefficients of determination *R*^2^ of the models are 0.9635 and 0.9539 for flexural strength and flexural modulus respectively (since the value is > 0.95). Therefore, the models are affirmed to be a good representation of the fitted data. The contributions of the inputs are 1.69 and 47.58% in the case of flexural strength while in the case of flexural modulus, the contribution values are 42.11 and 30.09%. Hence, particle sizes outperformed particle proportion regarding flexural strength. In the case of flexural modulus, particle proportion outperformed sizes. From the table, it is concluded by the linear interaction that each parameter can individually make appreciable shift in the response.

3 $$\begin{aligned} {\text{FS}} = & {18}.{5417} + {5}.{5543} {\text{A}} + 0.{685}0 {\text{B}} - 0.{1122} {\text{A}}*{\text{A}}{-}0.0{3275} {\text{B}}*{\text{B}} - 0.00{46} {\text{A}}*{\text{B}}, \\ {\text{R}}^{{2}} = & \, 0.{9635} \\ \end{aligned}$$

4 $$\begin{aligned} {\text{FM}} = & {1}.{4417} + 0.0{3478} {\text{A}}{-}0.0{3575} {\text{B}} - 0.000{8} {\text{A}}*{\text{A}} + \, 0.000{5} {\text{B}}*{\text{B}} - 0.000{1} {\text{A}}*{\text{B}}, \\ {\text{R}}^{{2}} = & \, 0.{9539} \\ \end{aligned}$$

**Table 5:** ANOVA result on flexural strength (FS).

Source	SS	df	MS	*F* value	*p* value	% Contribution
Model	3152.60	5	630.52	31.68	0.0003	96.35
*A*-Particle dosage	55.30	1	55.30	2.78	0.1466	1.69
*B*-Particle size	1556.82	1	1556.82	78.22	0.0001	47.58
AB	2.12	1	2.12	0.1063	0.7555	0.06
*A*^2^	1509.76	1	1509.76	75.86	0.0001	46.14
*B*^2^	28.60	1	28.60	1.44	0.2758	0.87
Residual	119.42	6	19.90			
Cor total	3272.02	11				

SS sum of squares, MS mean square.

**Table 6:** ANOVA result on flexural modulus (FM).

Source	SS	df	MS	*F* value	*p* value	% Contribution
Model	0.3652	5	0.0730	24.82	0.0006	95.39
*A*-particle dosage	0.1612	1	0.1612	54.77	0.0003	42.11
*B*-particle size	0.1152	1	0.1152	39.14	0.0008	30.09
AB	0.0017	1	0.0017	0.5742	0.4773	0.44
*A*^2^	0.0817	1	0.0817	27.75	0.0019	21.33
*B*^2^	0.0054	1	0.0054	1.83	0.2243	1.41
Residual	0.0177	6	0.0029			
Cor total	0.3828	11				

*SS* sum of squares, *MS* mean square.

Tables 7 and 8 displayed the analysis of variance for impact strength and hardness respectively. It is observed that the models are significant by virtue of the *p* value being less than 0.05. Particle dosage and size depicted consequential effect on the responses (*p* value is less than 0.05). The square interaction *A*^2^ is significant for impact strength response and inconsequential on hardness. Regarding the two responses, interactions AB and *B*^2^ are insignificant. The models (Eqs. 5 and 6) for the two responses are significant (*p* value is less than 0.05). Also, the coefficient of determination *R*^2^ is greater than 0.95 depicting the models have good correlation with fitted data. The contributions of the inputs are 49.95 and 14.35% in the case of impact strength while in the case of hardness, the respective 81.05 and 17.33%, indicating that particle proportion displayed higher contribution relative to particle sizes for the two responses. The linear model is more dominant than the interactions inferring the response parameters are showed more dependence on each liner term than their interactions.

5 $$\begin{aligned} {\text{IS}} = & {3}.{8771} + 0.0{517} {\text{A}}{-} \, 0.0{293} {\text{B}} - 0.00{14} {\text{A}}*{\text{A}} + 0.000{3} {\text{B}}*{\text{B}} + 0.0000 {\text{A}}*{\text{B}}, \\ {\text{R}}^{{2}} = & \, 0.{953}0 \\ \end{aligned}$$

6 $$\begin{aligned} {\text{HD}} = & {87}.{6667} + 0.{7633} {\text{A}} - 0.{475}0 {\text{B}} - 0.00{5}0 {\text{A}}*{\text{A}} + 0.00{25} {\text{B}}*{\text{B}} - 0.0000 {\text{A}}*{\text{B}} \\ {\text{R}}^{{2}} = & \, 0.{99}0{3} \\ \end{aligned}$$

**Table 7:** ANOVA result on impact strength (1S).

Source	SS	df	MS	*F* value	*p* value	% Contribution
Model	0.6876	5	0.1375	24.32	0.0006	95.30
*A*-particulate dosage	0.3604	1	0.3604	63.73	0.0002	49.95
*B*-particulate size	0.1035	1	0.1035	18.31	0.0052	14.35
AB	0.0001	1	0.0001	0.0111	0.9197	0.01
*A*^2^	0.2214	1	0.2214	39.15	0.0008	30.69
*B*^2^	0.0022	1	0.0022	0.3898	0.5554	0.31
Residual	0.0339	6	0.0057			
Cor total	0.7215	11				

*SS* sum of squares, *MS* mean square.

**Table 8:** ANOVA result on hardness (HD).

Source	SS	df	MS	*F* value	*p* value	% Contribution
Model	482.93	5	96.59	122.43	< 0.0001	99.03
*A*-Particulate dosage	395.27	1	395.27	501.04	< 0.0001	81.05
*B*-Particulate size	84.50	1	84.50	107.11	< 0.0001	17.33
AB	0.0000	1	0.0000	0.0000	1.0000	0.00
*A*^2^	3.00	1	3.00	3.80	0.0990	0.62
*B*^2^	0.1667	1	0.1667	0.2113	0.6620	0.03
Residual	4.73	6	0.7889			
Cor total	487.67	11				

*SS* sum of squares, *MS* mean square.

#### Surface and contour plot for responses

**Figure 5:**
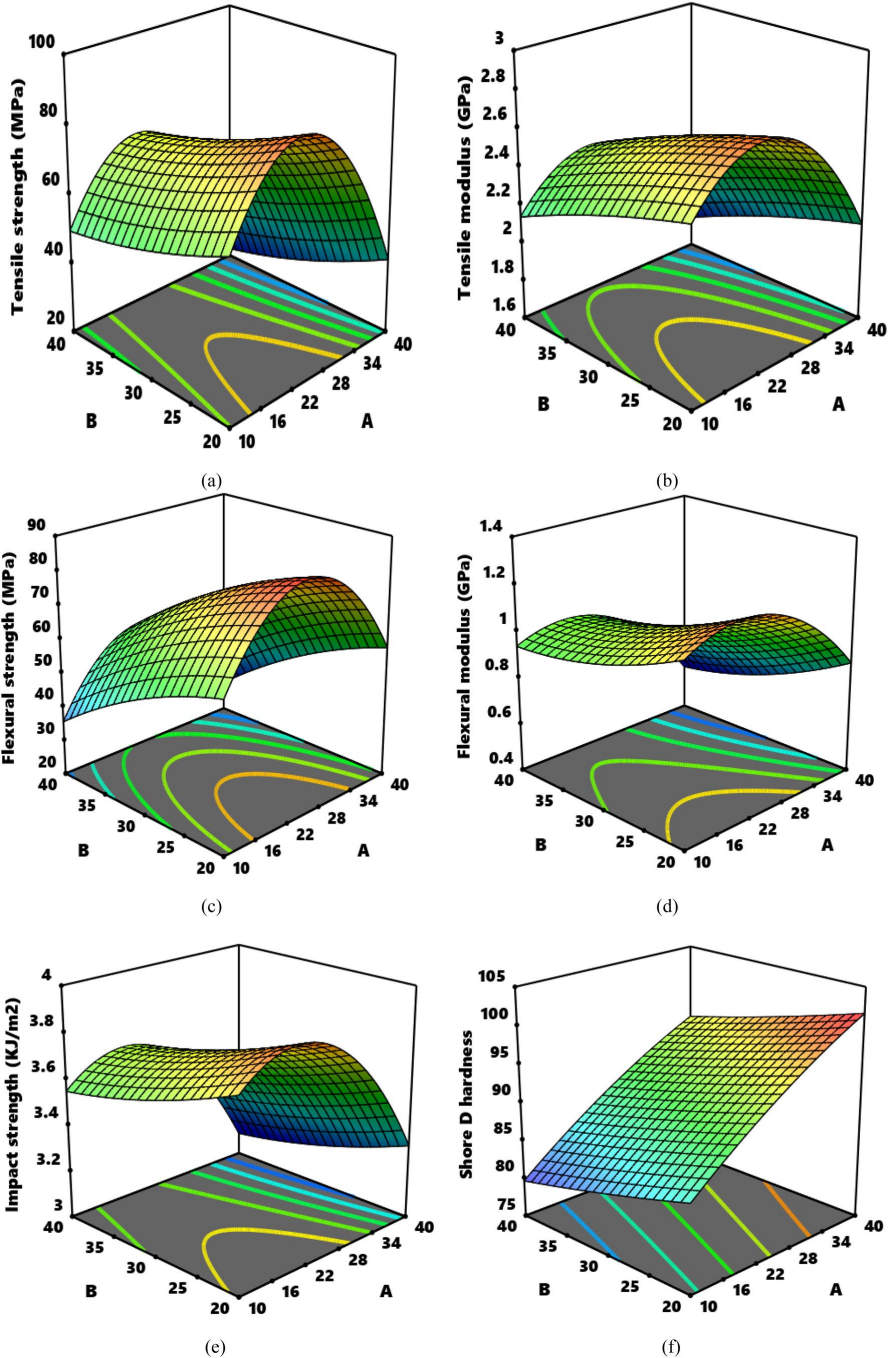
Surface plots for the relationship particulate proportion vs particulate size with respect (**a**) tensile strength (**b**) tensile modulus (**c**) flexural strength (**d**) flexural modulus (**e**) impact strength (**f**) hardness.** A** is particulate proportion,** B** is particulate sizes.

The effect of the interactions between the two independent variables: particulate proportion and particulate size on the responses is demonstrated in Fig. 5a,b,c,d,e,f. In Fig. 5a, it is portrayed that parameter A (particulate dosage) between 10 and 25.2059 wt.% showed increasing effect on tensile strength while proportions between 25.2059 and 40 wt.% is detrimental to the strength. From the same plot, increasing particulate size between 20 µm and 40 µm depicted depreciation in tensile strength. The plot points that optimum tensile strength is realizable when proportion is not above 25.2059 wt.% while particle size should be maintained at 20 µm. In Fig. 3(b), 10 to 27.75 wt.% particulate proportion depicted progressive enhancement in strength whereas particulate size exhibited negative linear pattern as there was gradual decrease in strength with increase in size. The surface plots for flexural strength and modulus displayed almost similar pattern for the interaction showing smiley inverted “U” shaped pattern. Particulate size in the range 10–27.2396 wt.% reflected increasing effect on flexural strength even as proportions beyond 27.2396 wt.% is detrimental to the strength. Equally, in the case of flexural modulus, dosage between 10 and 21.7656 wt.% is favourable for modulus enhancement, meanwhile, increasing particle size ensue progressive decrease in the response. As regards impact strength, particulate dosage between 10 and 20.0551 wt.% is synergetic to impact strength, beyond which particulate showed antagonistic effect on the response. In the case of hardness, increasing proportion of CSA led to linear improvement in hardness of the composite. Increment in size of the particles has negative consequence on hardness, as the value decreased with rise in proportion of CSA. The result had showed that each response depends more on the interactive dimension of each independent variables than their interactions. It therefore means each experimental factor can individually make appreciable shift in the responses.

**Figure 6:**
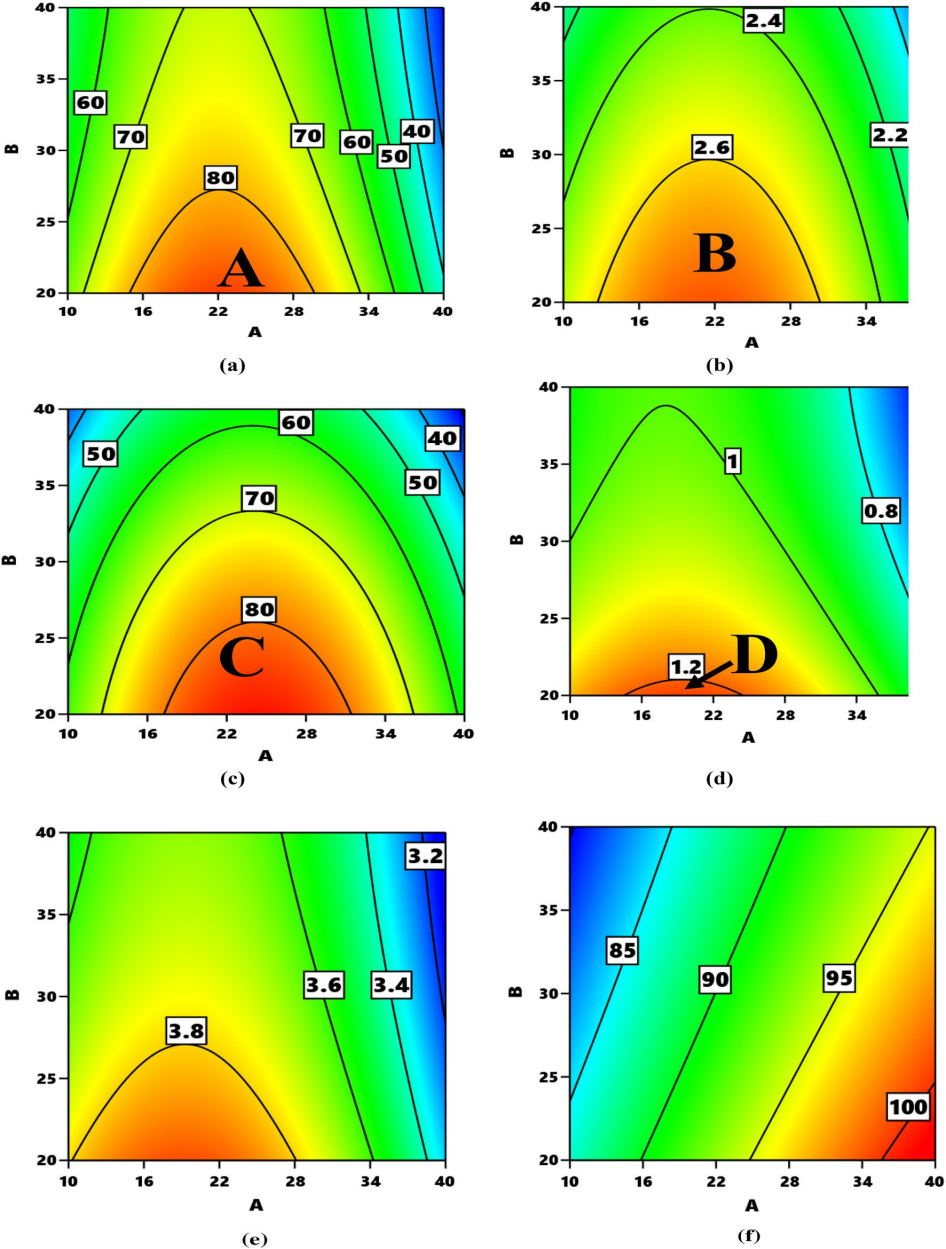
Contour plots for the relationship particulate proportion vs particulate size with respect to (**a**) tensile strength (**b**) tensile modulus (**c**) flexural strength (**d**) flexural modulus (**e**) impact strength (**f**) hardness.** A** is particulate proportion,** B** is particulate sizes.

The contour plots for the interactions between the two variables is represented in Fig. 6a–d. The areas marked A, B, C, and D, are the divisions of the plot where each response can be optimized respectively. The parametric combination of the variables in attaining optimizing each response parameter is depicted in Table 9.

**Table 9:** The parametric combination of the variables in attaining optimized response parameters.

Response	Marked optimum area	Range values for variables		Predicted range of responses	Predicted maximum value
		Proportion (wt.%)	Size (µm)		
Tensile strength	A	13.2–29.0	20–27.5	80–90 MPa	85.4298 MPa
Tensile modulus	B	11.8–29.8	20–29.5	2.6–2.8 GPa	2.6891 GPa
Flexural strength	C	17.0–30.5	20–26.2	80–90 MPa	82.5380 MPa
Flexural modulus	D	15.0–23.8	20–21.8	1.2–1.4 GPa	1.2121 GPa
Impact strength	E	10.2–28.1	20–27.5	3.8–4 KJ/m^2^	3.90158 KJ/m^2^
Hardness	F	35.7–40.0	20–24.7	100–105 HD	101.691 HD

#### Optimization for resultant responses

Further step was taken to optimize the overall mechanical properties as a single function utilizing the same software (design expert 13). The range for the goals and selected options for the optimization is indicated in Table 10.

**Table 10:** Goals and range for the optimization.

Name	Goal	Lower limit	Upper limit	Lower weight	Upper weight	Importance
A: CSA proportion	Is in range	10	40	1	1	3
B: Particle size	Is in range	20	40	1	1	3
TS	Maximize	21.8	92.3	1	1	3
TM	Maximize	1.76	2.83	1	1	3
FS	Maximize	30.6	86.6	1	1	3
FM	Maximize	0.63	1.26	1	1	3
IS	Maximize	3.15	3.97	1	1	3
HD	Maximize	80	101	1	1	3

The optimum condition attained via response surface analysis is indicated in Fig. 6.

**Figure 7:**
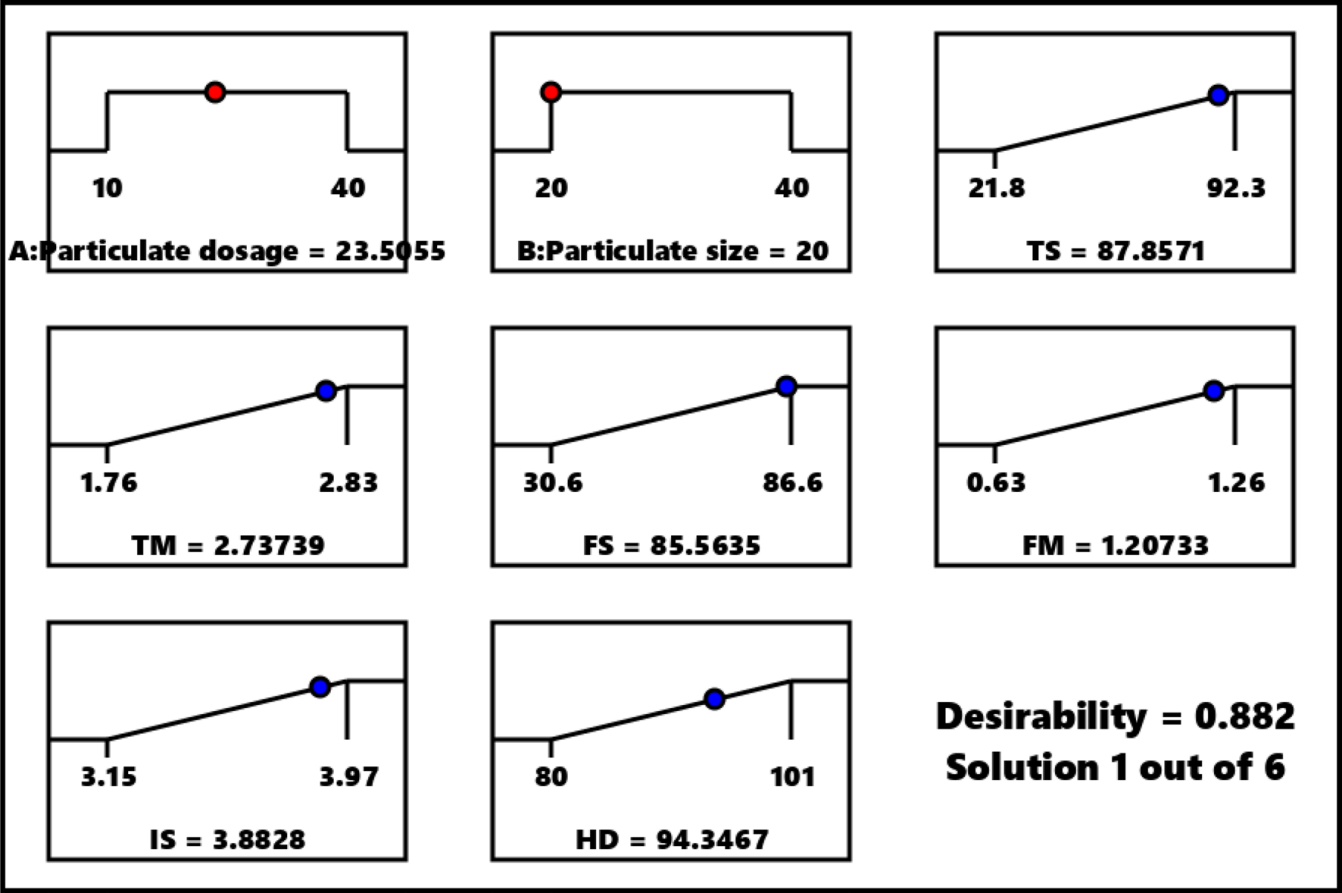
Optimization ramp for responses.

From Fig. 7, optimum condition is stipulated as 23.5055 wt.% and 20 µm for particulate proportion and particulate size respectively. Predicted responses at same condition are 87.8571 MPa, 2.7373 GPa, 85.5635 MPa, 1.20733 GPa, 3.8828 KJ/m^2^ and 94.3467 HD for tensile strength, tensile modulus, flexural strength, flexural modulus, impact strength and hardness in that order. Validated result was obtained as 90.49 MPa (+ 1.03% error), 2.68 GPa (− 2.0% error), 88.30 MPa (+ 3.2% error), 1.18 GPa (− 2.4% error), 4.03 KJ/m^2^ (+ 3.8% error) and 96.84 HD (+ 2.64% error) from the respective responses. Since the error is < 5%, the models are termed significant for predictions. Composite developed with optimum composition can find application in automobile for dashboard, seat and safety belts, seat cover, and airbags as well as other lightweight engineering applications.

### Microstructural analysis

**Figure 8:**
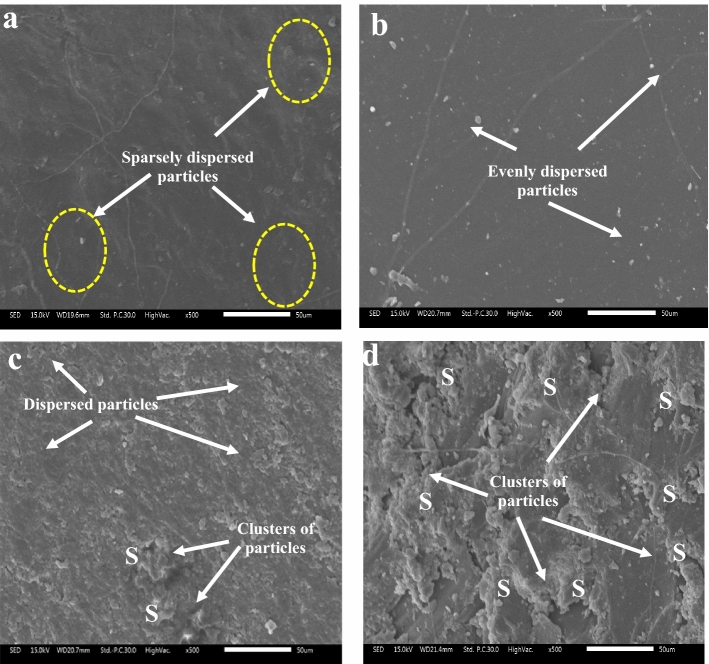
SEM images of selected specimen prepared with coconut shell ash of particle size 20 µm and proportions of (**a**) 10 wt.% (**b**) 20 wt.% (**c**) 30 wt.% and (**d**) 40 wt.%.

For the purpose of investigating the influence of the particles on the microstructure, the SEM images of the specimen (prepared with 20 µm CSA) was evaluated (Fig. 8). Figures 8a, b, c and d present the images of specimens prepared at 10, 20, 30, and 40 wt.% respectively. The CSA particles in Fig. 8a (for specimen doped with 10 wt.%) were indicated to be sparsely dispersed. The effect of the sparse dispersion was reflected in the sample's strength responses, which showed an improvement in all property parameters compared to the reference mix.

The SEM image of the specimen containing 20% CSA was highlighted in Fig. 8b. The particles were revealed to be evenly dispersed within the microstructure.

As a result of this, the composites' performance was improved when compared to the reference mix. In addition, when comparing the performance of specimens doped with 10% CSA with those doped with 20% CSA, the specimens doped with 20% CSA performed better in all properties measured. This occurs as a result of the particles filling inherent pores and acting as a barrier to the polymer molecules' movement. Furthermore, even dispersion gave the polymer chains a high degree of stiffness, resulting in increased strength.

Agglomeration, on the other hand, occurs at higher percentages of the fillers, as seen in Fig. 8c for a 30 wt.% CSA inclusion. The figure displayed two types of regions: particle dispersion regions and particle clusters regions labeled S. Clustering of particles has been shown to have a deleterious impact on the microstructure by acting as a stress concentration sites^69,70^. The region denoted by the letter S makes a further reference to this. This is an area of the matrix where residual strain is induced. As a result, a progressive residual defect develops inside the microstructure, consequently initiating and propagating cracks under loading. This defect is blamed for the reduced strength of specimens doped with 30 wt.% CSA compared to specimens doped with 20 wt.% CSA.

Regardless, when comparing the general performance of the specimen reinforced with 30 wt. % to the reference, the specimen reinforced with 30 wt. % outperforms the reference specimen. The discrepancy in performance could be due to the reference mix's greater pore volumes, which could facilitate easy crack initiation and propagation. Simply put, specimens containing 30 wt.% CSA outperformed the reference mix. This suggests that the favorable effect of particle dispersion in the 30 wt.% doped specimen outweighs the negative effect of agglomeration. This may be the case, as evidenced by the SEM image (Fig. 8c), which shows that cites of particle cluster have smaller ratios than cites of particle dispersion.

Because of the higher particle density in Fig. 8d, the image exposes more regions of particle clusters for particles doped with 40 wt.% CSA. As a result, a larger region of residual stress has been created inside the matrix, resulting in poor specimen performance. The mentioned point is blamed for the composite's poor performance at 40 wt.% CSA. The performance is inferior to those of other compositions and the matrix. As a result, the findings of this study reveal that particle proportions have a major impact in microstructural aspects.

## Conclusion

CSA particles of different proportion (10, 20, 30, and 40 wt.%) and particle sizes (40, 30, 20 µm) were used as filler in polyester matrix for lightweight engineering application. The mechanical properties were accessed. The following conclusions were made.

i. Proportion of 10 and 20 wt.% of the CSA resulted in enhancement of tensile strength. Meanwhile, 30 and 40 wt.% CSA induced strength decrease. With reduction in particle sizes from 40 to 20 µm, higher tensile strength was noted. Optimum tensile strength was attained at 20 wt.% CSA for all particle sizes.
ii. Tensile modulus increased as proportion of CSA increased from 10 to 40 wt.%. It was observed that lower particle size outperformed the larger particles.
iii. Flexural strength was improved on inclusion of 10 and 20 wt.% of the particles, of which beyond that there was progressive degradation of the strength. Lower particle sizes performed better than the higher particle sizes.
iv. Flexural modulus was enhanced with 10 and 20% CSA particle, but 30 to 40% engendered modulus reduction. Optimum flexural performance was attained at 20% CSA addition. It was noted that smaller particle sizes performed better than the larger sizes.
v. 10 and 20% CSA dosage spawned improvement of impact strength with 20 µm particle sizes yielding best performance. However, proportions beyond 20% triggered strength reduction
vi. Statistical analysis showed that the experimental variables; particulate proportion and size have significant contribution on the responses.
vii. The developed models were statistically fit as good representation of the experimental outcomes.

It was concluded that 20 µm CSA particles resulted into the optimum improvement of polyester matrix for lightweight applications.

## Data Availability

All data generated or analyzed during this study are included in this published article.
